# Prognostic Value and Potential Regulatory Mechanism of Alternative Splicing in Geriatric Breast Cancer

**DOI:** 10.3390/genes11020200

**Published:** 2020-02-16

**Authors:** Xin Li, Yaxuan Wang, Bingjie Li, Wang Ma

**Affiliations:** School of Medicine, Henan Polytechnic University, Jiaozuo 454150, Henan, China; Lixin9291@outlook.com (X.L.); wangyaxuan0216@outlook.com (Y.W.); Bingjie.li@monash.edu (B.L.)

**Keywords:** alternative splicing, geriatric breast cancer, splicing factors

## Abstract

Breast cancer has the highest mortality and morbidity among women, especially in elderly women over 60 years old. Abnormal alternative splicing (AS) events are associated with the occurrence and development of geriatric breast cancer (GBC), yet strong evidence is lacking for the prognostic value of AS in GBC and the regulatory network of AS in GBC, which may highlight the mechanism through which AS contributes to GBC. In the present study, we obtained splicing event information (SpliceSeq) and clinical information for GBC from The Cancer Genome Atlas, and we constructed a GBC prognosis model based on AS events to predict the survival outcomes of GBC. Kaplan–Meier analysis was conducted to evaluate the predictive accuracy among different molecular subtypes of GBC. We conducted enrichment analysis and constructed a splicing network between AS and the splicing factor (SF) to examine the possible regulatory mechanisms of AS in GBC. We constructed eight prognostic signatures with very high statistical accuracy in predicting GBC survival outcomes from 45,421 AS events of 10,480 genes detected in 462 GBC patients; the prognostic model based on exon skip (ES) events had the highest accuracy, indicating its significant value in GBC prognosis. The constructed regulatory SF–AS network may explain the potential regulatory mechanism between SF and AS, which may be the mechanism through which AS events contribute to GBC survival outcomes. The findings confirm that AS events have a significant prognostic value in GBC, and we found a few effective prognostic signatures. We also hypothesized the mechanism underlying AS in GBC and discovered a potential regulatory mechanism between SF and AS.

## 1. Introduction

Breast cancer, which has the highest mortality and morbidity rate among women in the world, has placed a heavy burden on global public health, especially in developing countries. According to GLOBOCAN 2018, 2.08 million new cases and 620,000 deaths due to breast cancer were reported in 2018, which accounted for 11.6% of all new cancer cases and 6.6% of cancer deaths of women [1]. With increasing age, the prevalence of breast cancer increases. In the United States, 43% of breast cancers are recognized in women aged older than 65 years. Age is undoubtedly the biggest hazard factor in breast cancer [2,3,4]. A statistically significant difference exists in the distribution of molecular subtypes between geriatric and young breast cancer patients, and less aggressive Luminal A and Luminal B tumor subtypes are more common in geriatric patients [5]. Previous studies have shown that estrogen receptor (ER) and progesterone receptor (PR) positives are higher in geriatric breast cancer (GBC), with less overexpression of epidermal growth factor receptor (EGFR), human epidermal growth factor receptor 2 (HER2), and ki67 [6]. At present, the effectiveness of prognostic signatures of breast cancers based on morphological classification and molecular biology is limited in predicting the overall survival outcomes of GBC patients, and most of these prognostic signatures are more applicable to young breast cancer patients, owing to the non-standard treatment and different molecular subtypes in GBC patients [7,8,9,10,11,12]. Therefore, novel and effective signatures for predicting the prognosis of GBC are urgently needed.

According to the Precision Medicine Initiative, precision medicine is a new method of medical treatment that is specified based on a patient’s genetics, environment, and lifestyle [13,14]. The development of genome sequencing has made precision medicine the core idea of current anti-cancer treatment. Therefore, studies have tried to seek trustworthy genetic changes from the perspective of alternative splicing (AS) to improve statistical accuracy in predicting GBC prognosis. AS plays a key role in the arrangement of protein diversity and gene expression of various eukaryotes. In humans, approximately 95% of multiple exon genes undergo AS [15,16]. Global analysis has revealed that at least 15,000 cancer-specific splice variants exist in 27 types of cancer. Cancer cells usually show abnormal AS profiles, which may be due to mutations at the splice sites (SS) or splicing regulatory elements [17,18]. Abnormal AS events may be associated in the development and advancement of cancer, including cell proliferation, apoptosis, invasion, tumor metastasis, angiogenesis, and metabolism [18,19].

Some studies have shown the association between AS events and breast cancer. For example, the increased risk of breast cancer metastasis is related to B-cell lymphoma-extra large (Bcl-xL) overexpression [20]. Abnormal splicing of ER and HER2 has been proven to promote breast carcinogenesis, which could be a feasible target for cancer treatment [21,22]. However, systematic analysis of the prognostic power of AS in GBC and the underlying mechanism is lacking. In general, AS is a sophisticated process that is strictly managed by the splicing factor (SF), and SFs are highly variable in terms of both function and framework [23]. The spliceosome is a highly sophisticated and dynamic ribonucleoprotein (RNP) machine, which is composed of the five small nuclear ribonucleoproteins (snRNPs) (U1, U2, U4, U5, and U6) and a large number of non-snRNP protein factors [24]. Studies have shown that the expression of SFs in cancer cells and normal tissues is significantly different, and mutations on SFs are closely associated with the occurrence of cancer [23,25]. Studies have shown that SF plays an important role in breast cancer hallmarks, such as angiogenesis, resisting cell death, sustaining proliferation, deregulating cellular energetics, and invasion and metastasis formation [26]. Therefore, studying the clinical significance of AS and SF in GBC and the potential regulatory mechanism pathways between them is valuable, as it may be the mechanism contributing to GBC.

The Cancer Genome Atlas (TCGA) database provides extensive genome data related to different cancers [27]. Many studies have employed TCGA splicing data to study AS events and their clinical significance associated with cancer, such as lung cancer, prostate cancer, gastrointestinal adenocarcinoma, bladder cancer, and ovarian cancer [28,29,30,31,32]. However, no study has fully investigated AS events and their prognostic value associated with GBC. Therefore, we aimed to use the data in TCGA database to construct a GBC prognosis model based on AS events to predict the survival outcomes of GBC. Kaplan–Meier (KM) analysis incorporating AS signature and molecular subtypes was used to verify the efficacy of prognosis models. Correlation analysis was employed to build a splicing network between AS and SF, in order to study the possible regulatory mechanisms of AS in GBC.

## 2. Materials and Methods

### 2.1. Process of Alternative Splicing Data Acquisition

TCGA SpliceSeq is a web-based resource that can provide data on AS events from 33 different tumor types (including available adjacent normal samples) [33]. The combination of gene symbol, splicing type, and splicing ID number constitutes the expression of each AS event. AS events are divided into seven types, including exon skip (ES), mutually exclusive exon (ME), retained intron (RI), alternate promoter (AP), alternate terminator (AT), alternate donor site (AD), and alternate acceptor site (AA). We obtained the percent-sliced-in (PSI) values for these seven types of AS events to quantify them in GBC. The PSI value ranges from 0 to 1, indicating that the AS event has changed. We also downloaded data of AS events of GBC from the TCGA SpliceSeq database.

We downloaded and extracted the clinical information about GBC from the pan-cancer atlas database of TCGA [34]. The clinical data of 1082 breast cancer patients were obtained from the TCGA database. We selected cases with a survival time greater than 90 days and age over 60 years as the GBC data (to exclude deaths that were not caused by the tumor), and we obtained 462 sets of data that met the requirements. The standard for non-GBC data is a survival time greater than 90 days and age less than 60 years.

### 2.2. A Preview of Survival-Related Alternative Splicing Events in Geriatric Breast Cancer

In this study, we included 462 GBC patients, and the overall survival (OS) was at least 90 days. Univariate Cox regression analysis was performed for every AS event, in order to screen AS events related to survival (*p* < 0.05). We used the UpSetR package in R to draw an UpSet diagram to show the set of interactions between seven types of survival-related AS events [35,36].

### 2.3. Prognostic Signatures for Alternative Splicing Events in Geriatric Breast Cancer

The least absolute shrinkage and selection operator (LASSO) method can reduce the dimensionality of high-dimensional data and obtain a better-fitting model. The LASSO Cox regression model was used to identify the ideal coefficients for each prognostic signature [37,38]. Multivariate Cox regression analysis was employed in the most important survival-related AS events that were selected from each AS type to establish a prognostic signature (PS). The AS events that were selected by the multivariate Cox regression analysis were used to determine the risk scores for the corresponding AS type: risk score = $\overset{n}{\underset{i}{\sum }}\mathrm{PSIi}\times \beta \;i$, where β is the regression coefficient in the multivariate Cox regression and PSI values are used to report alternative splicing changes. This was the calculation formula for the risk score of each splicing prognostic signature.

### 2.4. Evaluation of the Prognostic Value of the Risk Score

The clinical value of the risk score was evaluated using KM analysis and the receiver operating characteristic (ROC) curve. The median risk score was used to divide GBC patients into high-risk and low-risk groups; to further verify whether they had completely different prognoses, we performed a Kaplan–Meier analysis. Survival software was employed to calculate the estimated area under the curve (AUC) of the ROC curve to assess the predictive efficacy of each prognostic indicator in GBC [39]. Models with AUC > 0.7 were considered to be more effective models. Molecular subtypes are important factors influencing survival time. Therefore, the prognostic signatures were tested for their ability to predict the survival conditions of patients with different molecular subtypes using KM survival analysis. In this study, the molecular subtypes were divided into ER positive, ER negative, PR positive, PR negative, HER2 positive, HER2 negative, BRCA1 mutation, and BRCA1 non-mutation. BRCA1 had only five mutations; therefore, we could not perform survival analysis with the BRCA1 mutation subgroup. We placed non-GBC data into the prognostic signatures to evaluate the difference of AS events between GBC and non-GBC patients.

### 2.5. Building of the Potential Splicing Factor–Alternative Splicing Regulatory Network and Enrichment Analysis

SFs play an indispensable role in regulating the development and advancement of malignancy [23,25]. The information about the SFs was obtained from the database SpliceAid2 (which can be downloaded from http://www.introni.it/splicing.html) and previous studies [40,41,42]. The messenger RNA (mRNA) profiles of splicing factors in breast cancer and normal tissues were obtained from the TCGA database. Survival-related SFs were screened by univariate Cox regression analysis. Pearson correlation analysis was performed on differential expression of survival-associated SFs and corresponding independent prognostic AS events (screening criteria: |correlation coefficient| > 0.6, *p* < 0.001). Then, Cytoscape3.7.1 was used to establish the feasible regulatory network using the screened data [43]. Gene ontology (GO) terms and Kyoto Encyclopedia of Genes and Genomes (KEGG) pathways were used to assess the functions of the most important survival-related AS events. The most important pathways in KEGG and each GO category are visualized as shown.

## 3. Results

### 3.1. Information about Alternative Splicing Events

**Figure 1:**
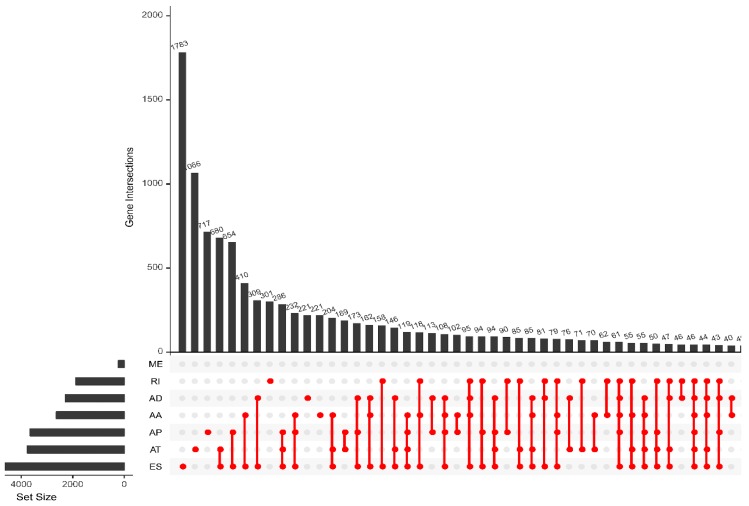
UpSet plots of splicing events in breast cancer. AA: alternate acceptor; AD: alternate donor; AP: alternate promoter; AT: alternate terminator; ES: exon skip; ME: mutually exclusive exons; RI: retained intron.

In general, we detected 45,421 AS events from 10,480 genes in breast cancer patients. These results include 3731 alternate acceptor (AA) events in 2628 genes, 3246 alternate donor (AD) events in 2278 genes, 9112 alternate promoter (AP) events in 3654 genes, 8595 alternate terminator (AT) events in 3755 genes, 17,702 exon skip (ES) events in 6811 genes, 233 mutually exclusive exon (ME) events in 227 genes, and 2802 retained intron (RI) events in 1878 genes (Table 1). The intersection set of genes and AS events is shown in the UpSet diagram in Figure 1. The total number of genes is much lower than the number of AS events, which indicates that a single gene may undergo more than one splicing. ES is the main type of AS event, and ME is rare.

In this study, to accurately describe an AS event, each AS event has a unique code. For example, for the code INO80B|54064|AA, INO80B is the gene name, *AA* is the splicing type, and 54,064 is the sequence number of the splicing event in the TCGA database.

### 3.2. Survival-Related Alternative Splicing Events

**Figure 2:**
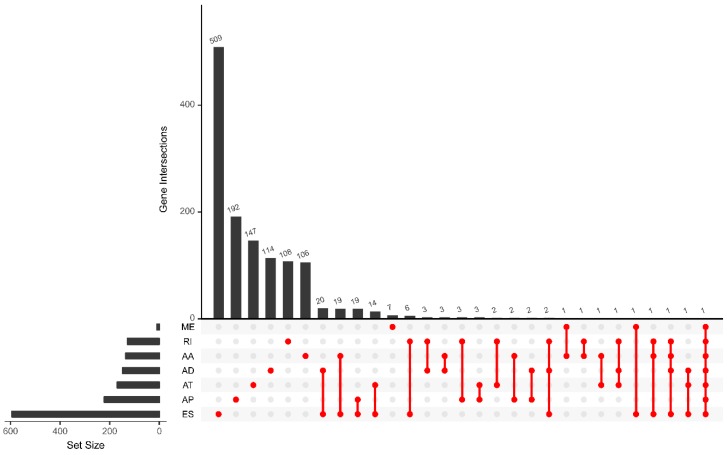
UpSet plots of prognosis-related alternative splicing (AS) events in geriatric breast cancer (GBC).

Through univariate Cox regression analysis, we identified a total of 1698 survival-associated AS events from 1289 genes in 462 GBC patients (*p* < 0.05), including 141 AA events in 136 genes, 158 AD events in 147 genes, 308 AP events in 221 genes, 247 AT events in 169 genes, 695 ES events in 593 genes, 9 ME events in 9 genes, and 140 RI events in 128 genes (Table 1). A gene may have two or more AS events that are prominently associated with the prognosis of GBC, so we used the Upset diagram to show the distribution of survival-related splicing events in the seven AS types and visualize the intersection set. The Upset diagram (Figure 2) clearly shows that ES is the most common event related to the prognosis of GBC.

**Figure 3:**
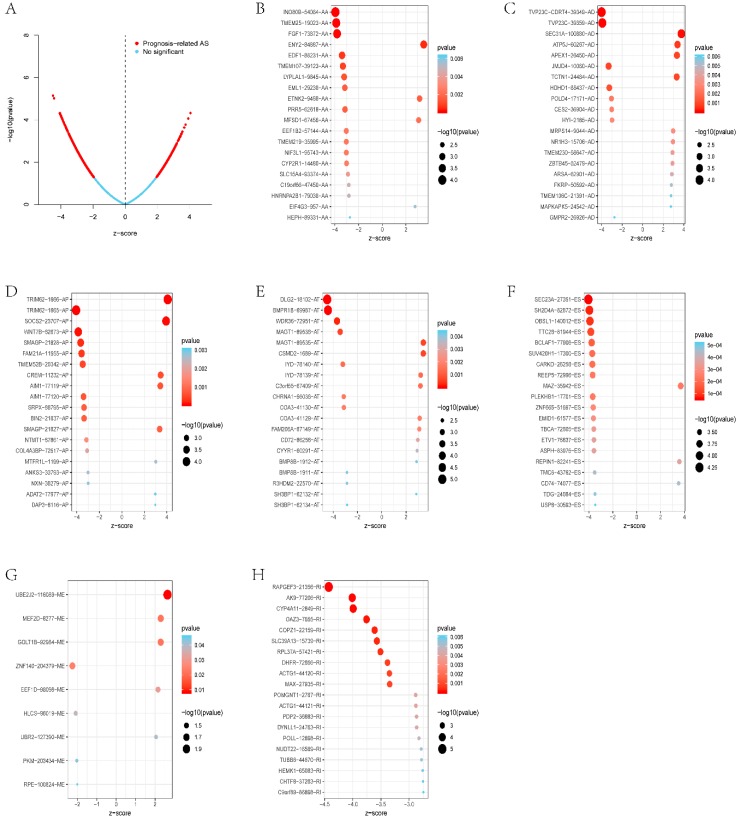
Most important AS events in GBC. (**A**) The red dots represent AS events that are prominently associated with patient survival. The blue dots represent AS events without correlation. (**B**) The top 20 AS events correlated with the clinical outcome based on alternate acceptors; (**C**) alternate donor sites; (**D**) alternate promoters; (**E**) alternate terminators; (**F**) exon skips; (**G**) the top nine AS events correlated with the clinical outcome, based on mutually exclusive exons; (**H**); retained introns.

The distribution of AS events related to survival is shown in Figure 3A. Figure 3B–H shows the 20 most important prognostic-related AS events. However, among ME events, there were only nine prognostic-related AS events.

### 3.3. Prognostic Signatures for Alternative Splicing Events in Breast Cancer

**Figure 4:**
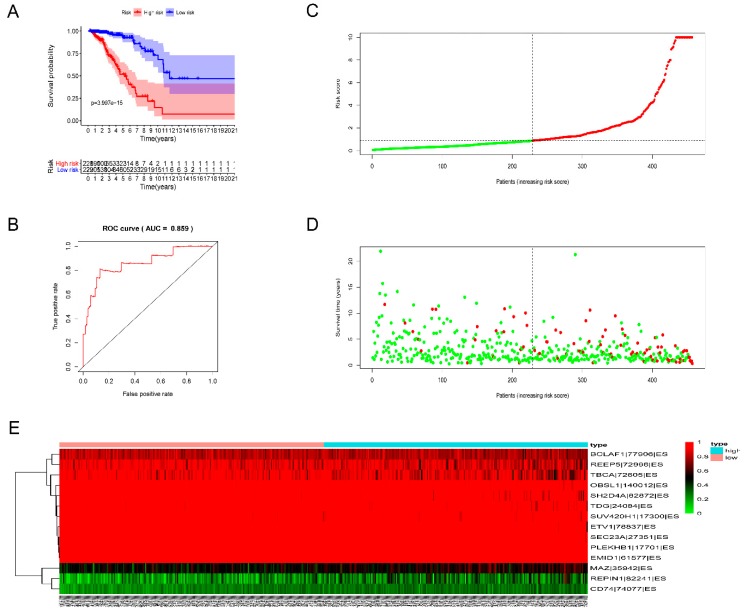
Analysis of the prognostic signature (PS)–exon skip (ES). The 14 prognosis-associated events of ES were selected by multivariate Cox regression analysis to make the PS–ES model. (**A**) Kaplan–Meier (KM) curves of prognostic signature built with ES events. (**B**) The receiver operating characteristic (ROC) curves of prognostic signatures constructed with ES events (**C**). Risk scores of GBC patients constructed by ES events. (**D**) Survival conditions and survival time of GBC patients, distributed according to risk score (green dots represent survivors, red dots represent deaths). (**E**) Heat map indicating the correlation between the percent-spliced-in (PSI) value of the ES events and the risk score. Colors from red to blue means decreasing PSIs from high to low.

**Figure 5:**
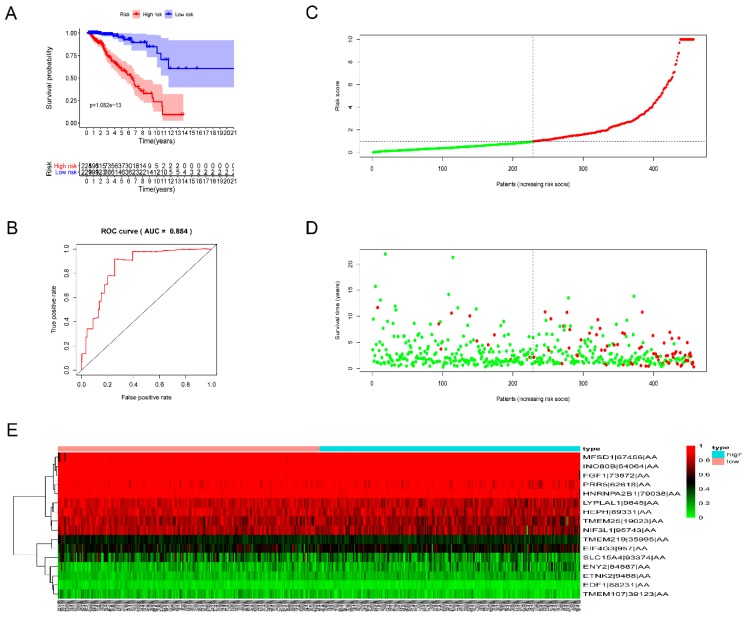
Analysis of the PS–alternate acceptor site (AA). The 16 prognosis-associated events of AA were selected by multivariate Cox regression analysis to make the PS–AA model. (**A**) Kaplan–Meier curves of prognostic signature built with AA events. (**B**) ROC curves of prognostic signatures construct with AA events. (**C**) Risk scores of GBC patients constructed by AA events. (**D**) Survival conditions and survival time of GBC patients, distributed according to risk score (green dots represent survivors, red dots represent deaths). (**E**) Heat map indicating the correlation between the PSI value of the AA events and the risk score. Colors from red to blue means decreasing PSIs from high to low.

**Figure 6:**
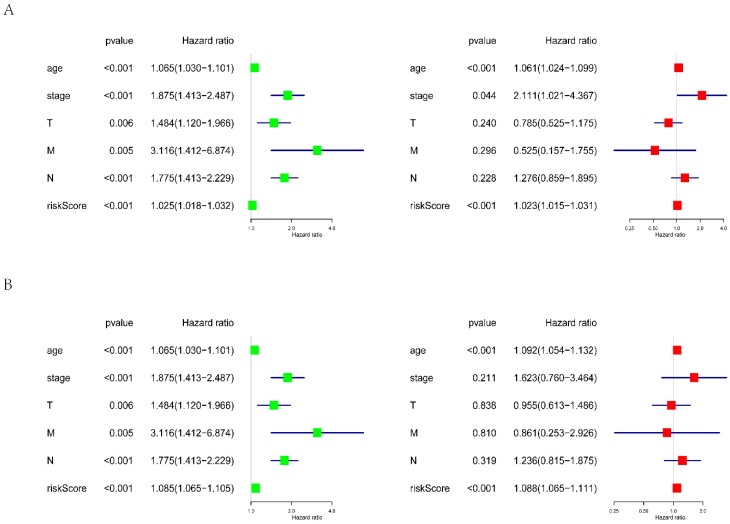
Univariate (left) and multivariate (right) independent prognostic analysis by Cox regression. Green dots represent the hazard ratio of different clinical parameters in univariate regression analysis, and red dots represent the hazard ratio of different clinical parameters in multivariate regression analysis. Independent prognostic analysis based on (**A**) a PS–ES model and (**B**) a PS–AA model. T: tumor; M: metastasis; N: lymph node.

We built prognostic signatures based on AA, AD, AP, AT, ES, ME, RI, and all types of AS events using LASSO Cox analysis after univariate Cox to eliminate interacting genes after cross-validation of the minimum error (Figure S1A–H) and screen significant survival-associated genes (Figure S1I–P). Figure S2 shows the distribution of percent-spliced-in (PSI) values and risk scores in each prognostic signature. All prognostic signatures showed that higher risk scores lead to higher mortality. We evaluated the predictive efficiency of the models through KM analysis and ROC curves (Figures S3 and S4). The risk score was calculated according to the above method, and then the median risk score was used to divide GBC patients into high-risk and low-risk groups. KM analysis (Figure S3) showed that for all prognostic signatures, the survival time of GBC patients in the high-risk group was significantly less than that in the low-risk group (*p* < 0.001). These results suggest that the pronounced molecular characteristics of AS events are adverse prognostic factors in GBC. Survival ROC analysis was performed to compare the predictive power of every prognostic signature (Figure S4). The data showed that the AUC values of AA, AD, AP, AT, ES, ME, RI, and all AS models were 0.854, 0.738, 0.84, 0.764, 0.859, 0.707, 0.835, and 0.785, respectively. The prognostic signature of ES (Figure 4) shows the best predictive efficiency, followed by the AA model (Figure 5). The ES model has a great potential for predicting the survival of GBC patients. According to the evaluation of univariate and multivariate Cox regression analysis, the comprehensive analysis results showed that only the age and the risk score of the eight prognostic models have independent significant prognostic value compared with other clinical parameters, including age, cancer stage, and tumor T, N, and M stages (*p* < 0.01) (Figure 6, Table 2).

By substituting data from non-GBC patients into established prognostic signatures, the survival time of non-GBC patients in the low-risk group and the high-risk group did not show a significant difference; even the survival time of the low-risk group was less than that in the high-risk group in the PS–AT group (Figure S5). The KM analyses of all prognostic signatures showed that the high-risk group had shorter survival times than the low-risk group in the cohort classified by Her-2 status, ER status, PR status, and BRCA1 status (Figures S6–S13).

### 3.4. Survival-Associated Potential of the Splicing Factor–Alternative Splicing Regulatory Network and Enrichment Analysis

**Figure 7:**
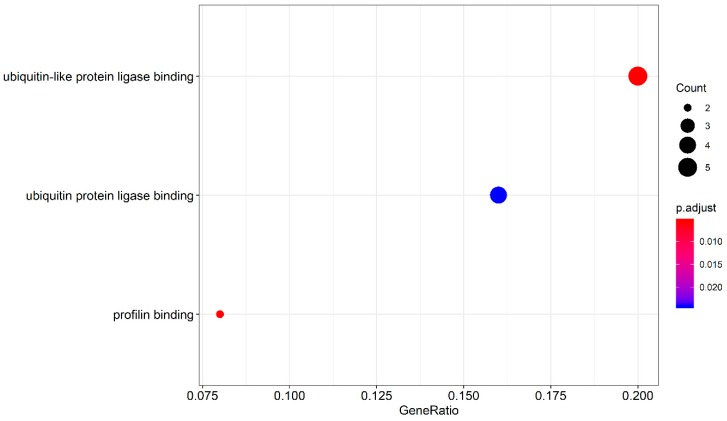
Pathways identified by Gene Ontology (GO) analysis. The genes from survival-associated AS events in GBC were subjected to GO pathway analyses. The dot size stands for the number of the enriched genes, and FDR values are shown by the color scale. FDR: false discovery rate.

**Figure 8:**
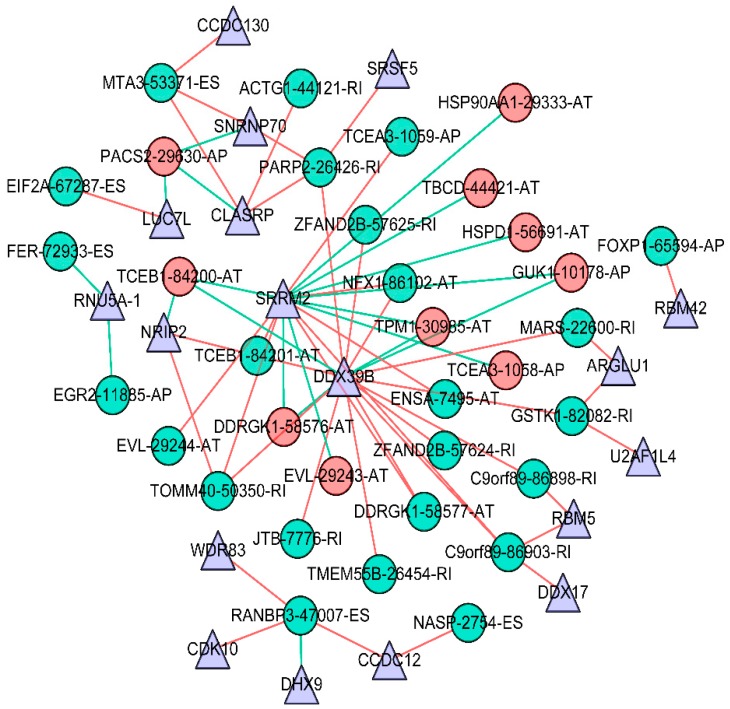
Survival-associated potential SF–AS regulatory network in breast cancer. Triangles (*n* = 18) represent SFs, red circles represent AS events associated with poor prognosis, green circles represent AS events associated with favorable prognosis, red lines indicate a positive regulation between AS and SF, and green lines indicate a negative regulation between AS and SF.

SFs play an important role in regulating the occurrence of splicing events. These SFs, usually bound to pre-mRNA, regulate splicing by affecting exon selection and splicing sites, which are closely associated with the development and progression of tumors. We downloaded the information on SFs from the SpliceAid2 database, as well as previous studies (Table S1). Pearson correlation analysis was employed to study the relationship between the differential expression of survival-associated SFs and independent prognostic AS events (screening criteria: |correlation coefficient| > 0.06, *p* < 0.001). We found 18 SFs and 34 AS-related independent prognostic events that were apparently associated with the prognosis of GBC patients. These significant, survival-related AS events were used to investigate enrichment in biological functions and pathways (Figure 7). The GO analyses showed that the prominent survival-related AS events were clustered in biological processes, including ubiquitin-like protein ligase binding and profilin binding (*p* < 0.01), and that KEGG analysis did not identify useful pathways. Then, the data obtained from the correlation analysis were introduced into Cytoscape 3.7.1 to establish the AS–SF correlation network (Figure 8). In this network, triangles represent SFs, red circles represent AS events associated with poor prognosis, green circles represent AS events associated with favorable prognosis, the red lines represent a positive regulation between AS and SF, and green lines represents a negative regulation between AS and SF. Different AS events in SF may have different functions. For example, DDX39B has a positive correlation with DDRGK1-58577-AT but a negative correlation with DDRGK1-58576-AT. Notably, AS events associated with poor prognosis are mainly negatively correlated with SF, whereas AS events associated with favorable prognosis are mainly positively correlated. The relationship between SF and AS is not one-to-one. An SF can be concerned with multiple independent prognostic AS events, and an independent prognostic AS event also can be concerned with multiple SFs.

## 4. Discussion

Violating the “one gene, one polypeptide” rule, AS exerts strong effects on gene expression by producing multiple protein isoforms. AS can cause the generated mRNA to be degraded by nonsense-mediated mRNA decay, ultimately changing the quality and quantity of protein products [44,45]. Studies have shown that abnormal AS events may be the mechanism underlying the processes of different diseases, including the occurrence and development of tumors [18,19]. Cancer-specific mRNA produced by abnormal AS events may cause the dysfunction of tumor suppressors or activation of oncogenes, which participate in the development of tumors. [46,47]. More and more studies are recognizing the relationship between abnormal AS events and tumors. For example, SNRPB is currently considered a prognostic marker for glioblastoma [48]. Out-of-control AS events regulated by SRSF1 can encourage the formation of breast cancer [49]. However, comprehensive and scientific analysis of the prognostic power of AS events in GBC is lacking. To the best of our knowledge, this is the first study to use the TCGA database to integrate AS events and clinical factors to comprehensively study the prognostic significance of AS events in GBC. We constructed eight prognostic signatures based on AA, AT, AD, AP, ES, ME, RI, and all types of AS events, with significant predictive power for the overall survival of GBC patients. Among them, the ES model showed the best predictive performance (AUC = 0.859). Through univariate and multivariate Cox analysis, we discovered that only the age and risk score of the eight prognostic models had independent significant prognostic value. KM analysis was also used to investigate the survival outcomes of the different molecular subgroups of GBC. We established a potential AS–SF regulatory network, and pathway and process enrichment analyses were used to analyze the important survival-related AS events, which may be the mechanisms through which AS contributes to GBC.

The most important clinical significance of this study is the establishment of prognostic signatures that have significant predictive power. In previous studies, some researchers have developed prognostic models for breast cancer based on other genomic characteristics. For example, Zhang et al. established a prognostic model for breast cancer based on rapacity-regulated gene expression characteristics [50]. By studying the expression status of the *TP53* gene and autophagy genes, prognostic signatures have also been successfully established [51,52]. However, in our study, our main emphasis was not the impact of abnormal AS events of a certain gene on the prognosis of breast cancer patients; thus, we established prognostic signatures based on the system analysis of GBC-related AS events. KM analysis proved that all the prognostic signatures could accurately predict the survival outcomes of GBC in different kinds of molecular subtypes, including ER positive, ER negative, PR positive, PR negative, HER2 positive, HER2 negative, and BRCA1 non-mutation. According to our results, the eight prognostic signatures all have excellent clinical value, and the ES model was the best (AUC = 0.859), which could provide efficient prognostic value. However, when we placed non-GBC data into the prognostic signatures, the models that accurately predicted GBC showed no statistical significance, which confirms that the AS events are significantly different between GBC and non-GBC. These age-dependent variations in patient prognosis may be due to the BRCA1-driven differences and micro-environmental changes. In the ES model, we analyzed 14 AS events related to the prognosis of GBC, including ETV1, SH2D4A, BCLAF1, and SUV420H1. One breast cancer study indicated that cell proliferation and invasion in triple-negative breast cancer can be suppressed through miR-17-5p targeting ETV1, and ETV1 was proven to be a significant oncogene in triple-negative breast cancer [53]. This is also consistent with our findings; the overexpression of ETV1 is associated with poor prognosis. Other genes, such as *SH2D4A*, *BCLAF1*, and *SUV420H*, also have carcinogenic or tumor-suppressing functions that can affect the formation and development of human cancer [54,55]. Therefore, our findings may provide a new perspective for administering precision medicine and elucidating the molecular mechanisms underlying GBC tumorigenesis. AS is a complicated system that is strictly regulated by SFs [23]. Therefore, SFs are the key factor in adjusting splicing events, and the correlation between SFs and independent prognostic AS events is also worth studying.

Abnormal splicing of ER, HER2, CEACAM1, and SRSF1 has been reported to contribute to breast tumorigenesis and prognosis, which could be an underlying target for cancer treatment [21,22,49,56]. However, information is lacking about how AS and SF contribute to GBC. In this study, the regulatory network explained the potential regulatory mechanism between AS and SF in GBC (*p* < 0.001), which may explain how AS contributes to GBC. Negative correlations between SF and AS events in breast cancer were more common than positive correlations, and a single SF might play a dual role in different AS events: positive regulation or negative regulation. For example, DDX39B negatively regulated DDRGK1-58576-AT, whereas it positively regulated DDX39B DRGK1-58577-AT. Different SFs for the same AS event usually have a synergistic effect, but there are special cases. For example, RANBP3-47007-ES is under the positive regulation of CCDC12 and CDK10, but under the negative regulation of DHX9. Notably, AS events associated with poor prognosis were mainly negatively correlated with the SF, whereas AS events associated with favorable prognosis were mainly positively correlated with it. These results suggest that SFs and AS are not in a one-to-one relationship, and complex regulatory mechanisms exist between them.

The SF–AS regulatory network may provide a new direction for the underlying regulatory mechanisms. Thus, SRRM2 and DDX39B occupy an important position in the SF–AS regulatory network. According to prior studies, SRRM2 plays an important role in precise chromosome segregation, genome stability, and cell proliferation [57]. A germline mutation in SRRM2 is related to the predisposition of papillary thyroid carcinoma [58]. DDX39B promotes cell proliferation by up-regulating pre-ribosomal RNA, and its levels are apparently improved in various cancer types [59]. However, no previous study has discussed the effect of SRRM2 and DDX39B in GBC. Our results provide new directions for tumorigenesis in GBC.

The GO analysis results in our study indicate that the genes are mainly involved in pathways and biological processes, including the ubiquitin-like protein ligase binding and profilin binding. Protein ubiquitination is one of the most important posttranslational modifications of protein—it controls many cellular processes, including DNA damage response, cell cycle control, and cellular signaling [60]. Overexpression of Profilin-1 also has the ability to suppress the invasiveness and motility of breast cancer cells, which is a negative regulator of mammary carcinoma aggressiveness [61]. AS events produced by these genes might influence the development of GBC through participating in the above biological pathways and processes. In summary, we collated prognostic-related AS events in GBC through the TCGA database and established prognostic signatures with satisfactory predictive power in GBC. To reveal the regulatory mechanism of AS events contributing to GBC, we also established an SF–AS regulatory network and analyzed enrichment, which not only provides possible new prognostic indicators for GBC patients, but also may provide directions for further exploration of splicing-related mechanisms.

## Figures and Tables

**Table 1:** Overview of the splicing events in GBC.

Type	Total Splicing Events		SSEs	
	Splicing Events	Genes	Splicing Events	Genes
**AA**	3731	2628	141	136
**AD**	3246	2278	158	147
**AP**	9112	3654	308	221
**AT**	8595	3755	247	169
**ES**	17,702	6811	695	593
**ME**	233	227	9	9
**RI**	2802	1878	140	128
**Total**	45,421	21,231	1698	1403

SSEs: survival-associated splicing events. AA: alternate acceptor; AD: alternate donor; AP: alternate promoter; AT: alternate terminator; ES: exon skip; ME: mutually exclusive exons; RI: retained intron.

**Table 2:** Univariate and multivariate Cox regression analysis of eight PS models and clinical indexes of GBC patients.

Clinical Variable	Univariate		Multivariate	
	HR (95% CI)	*p*-Value	HR (95% CI)	*p*-Value
Age	1.064 (1.030–1.101)	0.0002	1.079 (1.042–1.117)	1.65 × 10^−5^
Stage	1.875 (1.413–2.487)	1.31 × 10^−5^	1.690 (0.809–3.529)	0.1627
T	1.484 (1.120–1.966)	0.006	0.887 (0.582–1.351)	0.557
M	3.116 (1.412–6.874)	0.0049	0.621 (0.192–2.009)	0.426
N	1.775 (1.413–2.229)	8.00 × 10^−7^	1.427 (0.946–2.153)	0.09
PS–AA	1.085 (1.065–1.105)	2.88 × 10^−18^	1.088 (1.065–1.111)	6.15 × 10^−15^
PS–AD	1.132 (1.102–1.1164)	3.28 × 10^−19^	1.133 (1.098–1.169)	6.92 × 10^−15^
PS–AP	1.043 (1.032–1.055)	3.54 × 10^−14^	1.041 (1.029–1.054)	3.41 × 10^−11^
PS–AT	1.011 (1.006–1.016)	1.13 × 10^−5^	1.012 (1.007–1.017)	4.79 × 10^−6^
PS–ES	1.025 (1.018–1.032)	1.87 × 10^−12^	1.023 (1.015–1.031)	2.87 × 10^−8^
PS–ME	1.502 (1.309–1.724)	6.79 × 10^−9^	1.546 (1.343–1.778)	1.08 × 10^−9^
PS–RI	1.011 (1.007–1.015)	9.69 × 10^−8^	1.011 (1.007–1.015)	5.22 × 10^−8^
PS–ALL	1.006 (1.003–1.009)	0.0002	1.006 (1.003–1.009)	4.87 × 10^−5^

HR:hazard ratio; T: tumor; M: metastasis; N: lymph node; PS–AA: prognostic signature–alternate acceptor; PS-AD: prognostic signature– alternate donor; PS–AP: prognostic signature–alternate promoter; PS–AT: prognostic signature– alternate terminator; PS–ES: prognostic signature– exon skip; PS–ME: prognostic signature– mutually exclusive exons; PS–RI: prognostic signature–retained intron; PS–ALL: prognostic signature–all types of AS events;.

## Supplementary Materials

The following are available online at https://www.mdpi.com/2073-4425/11/2/200/s1, **Figure S1:** Construction of prognostic signatures based on least absolute shrinkage and selection operator (LASSO) Cox analysis. (**A**–**H**): the lowest point of the ordinate is the minimum point of the cross-validation error (**A**); alternate acceptor (**B**); alternate donor sites (**C**); alternate promoters (**D**); alternate terminators (**E**); exon skips (**F**); exclusive exons (**G**); retained introns (**H**), all types. (**I**–**P**): LASSO Cox analysis of seven types of events. The horizontal axis stands for the Log Lambda. The vertical axis stands for the coefficients. As the value of the logλ increased, the coefficient approaches 0. (**I**) Alternate acceptors (**J**), alternate donor sites (**K**), alternate promoters (**L**), alternate terminators (**M**), exon skips (**N**), exclusive exons (**O**), retained introns (**P**), all types. **Figure S2.** Distribution of PSI values and risk scores in each PS model. The top of each diagram is the risk score curve of patients with breast cancer, the middle part represents survival status and survival time of breast cancer patients, distributed by risk score (green dots represent survivors, red dots represent the dead), and the bottom part shows the heat map of the PSIs. Colors from blue to red means increasing PSIs from low to high. (A) Risk scores constructed by significant survival-associated AS events in the AA type. (B) Risk scores constructed by significant survival-associated AS events in the AD type (C). Risk scores constructed by significant survival associated AS events in the AP type. (D) Risk scores constructed by significant survival-associated AS events in the AT type. (E) Risk scores constructed by significant survival-associated AS events in the ES type. (F) Risk scores constructed by significant survival-associated AS events in the ME type. (G) Risk scores constructed by significant survival associated AS events in the RI type. (H) Risk scores constructed by significant survival-associated AS events in all types. **Figure S3.** Kaplan–Meier curves of prognostic signatures for breast cancer. (A) Kaplan–Meier survival curves for the PS–AA model (p = 1.082e−13). (B) Kaplan–Meier survival curves for the PS–AD model (p = 3.464e−13). (C) Kaplan-Meier survival curves for the PS–AP model (p = 4.341e−09). (D) Kaplan–Meier survival curves for the PS–AT model (p = 3.44e−10). (E) Kaplan–Meier survival curves for the PS–ES model (p = 3.997e−15). (F) Kaplan–Meier survival curves for the PS–ME model (p = 9.415e−05). (G) Kaplan–Meier survival curves for the PS–RI model (p = 1.453e−10). (H) Kaplan–Meier survival curves for the PS–ALL model (p = 3.331e−16). **Figure S4.** ROC curves of prognostic signatures for breast cancer. (A) ROC curve for the PS–AA model (AUC = 0.854). (B) ROC curve for the PS–AD model (AUC = 0.738). (C) ROC curve for the PS–AP model (AUC = 0.84). (D) ROC curve for the PS–AT model (AUC = 0.764). (E) ROC curve for the PS–ES model (AUC = 0.859). (F) ROC curve for the PS–ME model (AUC = 0.707). (G) ROC curve for the PS–RI model (AUC = 0.835). (H) ROC curve for the PS–ALL model (AUC = 0.785). **Figure S5:** Non-GBC data substitutes into the PS model: (A) PS–AA model, (B) PS–AD model, (C) PS–AP model, (D) PS–AT model, (E) PS–ES model, (F) PS–ME model, (G) PS–RI model, (H) PS–All model. **Figure S6.** Kaplan–Meier curves of the PS–AA model for different molecular types of GBC patients. (A) ER negative, (B) ER positive, (C) HER2 negative, (D) HER2 positive, (E) PR negative, (F) ER positive, (G) BRCA1 negative. **Figure S7.** Kaplan–Meier curves of the PS–AD model for different molecular types of GBC patients. (A) ER negative, (B) ER positive, (C) HER2 negative, (D) HER2 positive, (E) PR negative, (F) ER positive, (G) BRCA1 negative. **Figure S8**. Kaplan–Meier curves of the PS–AP model for different molecular types of GBC patients. (A) ER negative, (B) ER positive, (C) HER2 negative, (D) HER2 positive, (E) PR negative, and (F) ER positive, (G) BRCA1 negative. **Figure S9.** Kaplan–Meier curves of the PS–AT model for different molecular types of GBC patients. (A) ER negative, (B) ER positive, (C) HER2 negative, (D) HER2 positive, (E) PR negative, (F) ER positive, and (G) BRCA1 negative. **Figure S10.** Kaplan–Meier curves of the PS–ES model for different molecular types of GBC patients. (A) ER negative, (B) ER positive, (C) HER2 negative, (D) HER2 positive, (E) PR negative, (F) ER positive, and (G) BRCA1 negative. **Figure S11.** Kaplan–Meier curves of the PS–ME model for different molecular types of GBC patients. (A) ER negative, (B) ER positive, (C) HER2 negative, (D) HER2 positive, (E) PR negative, (F) ER positive, and (G) BRCA1 negative. **Figure S12.** Kaplan–Meier curves of the PS–RI model for different molecular types of GBC patients. (A) ER negative, (B) ER positive, (C) HER2 negative, (D) HER2 positive, (E) PR negative, (F) ER positive, and (G) BRCA1 negative. **Figure S13.** Kaplan–Meier curves of the PS–ALL model for different molecular types of GBC patients. (A) ER negative, (B) ER positive, (C) HER2 negative, (D) HER2 positive, (E) PR negative, (F) ER positive, and (G) BRCA1 negative. **Table S1:** Collection of 610 splicing factors from the SpliceAid 2 database and previous studies.
